# Members of the barley NAC transcription factor gene family show differential co-regulation with senescence-associated genes during senescence of flag leaves

**DOI:** 10.1093/jxb/eru046

**Published:** 2014-02-24

**Authors:** Michael W. Christiansen, Per L. Gregersen

**Affiliations:** Department of Molecular Biology and Genetics, AU-Flakkebjerg, Forsøgsvej 1, DK-4200 Slagelse, Denmark

**Keywords:** Barley, cereals, DNA microarray, gene expression, leaf senescence, NAC transcription factor, nutrient remobilization.

## Abstract

The senescence process of plants is important for the completion of their life cycle, particularly for crop plants, it is essential for efficient nutrient remobilization during seed filling. It is a highly regulated process, and in order to address the regulatory aspect, the role of genes in the NAC transcription factor family during senescence of barley flag leaves was studied. Several members of the NAC transcription factor gene family were up-regulated during senescence in a microarray experiment, together with a large range of senescence-associated genes, reflecting the coordinated activation of degradation processes in senescing barley leaf tissues. This picture was confirmed in a detailed quantitative reverse transcription–PCR (qRT–PCR) experiment, which also showed distinct gene expression patterns for different members of the NAC gene family, suggesting a group of ~15 out of the 47 studied NAC genes to be important for signalling processes and for the execution of degradation processes during leaf senescence in barley. Seven models for DNA-binding motifs for NAC transcription factors were designed based on published motifs, and available promoter sequences of barley genes were screened for the motifs. Genes up-regulated during senescence showed a significant over-representation of the motifs, suggesting regulation by the NAC transcription factors. Furthermore, co-regulation studies showed that genes possessing the motifs in the promoter in general were highly co-expressed with members of the NAC gene family. In conclusion, a list of up to 15 NAC genes from barley that are strong candidates for being regulatory factors of importance for senescence and biotic stress-related traits affecting the productivity of cereal crop plants has been generated. Furthermore, a list of 71 senescence-associated genes that are potential target genes for these NAC transcription factors is presented.

## Summary

Members of the NAC transcription factor family in barley appear to be highly involved in initiation and progression of leaf senescence via regulation of target genes having palindromic NAC-binding sequences in their promoters.

## Introduction

Plant nutrients are to a very large extent taken up and stored temporarily in the vegetative parts of cereal crop plants, either as structural components or as part of compounds in metabolic fluxes, until the seed filling and maturation stage, where they are remobilized to varying extents and translocated to the developing seeds. This developmental process is of the utmost importance for the productivity of crop plants, and a crucial part of it is the terminal process described as leaf senescence, in which a highly regulated degradation of the components of leaf tissues takes place (for reviews, see Buchanan-Wollaston *et al.*, 2003; Gregersen *et al.*, 2008; Gregersen, 2011). Senescence of plants is initiated in individual distal or lower organs and tissue parts, successively building up to whole-plant senescence (Noodén, 1988) which only specialized tissues in the reproductive organs survive. The monocarpic cereal crop plant constitutes a typical example of this process for which the only surviving tissues are the embryo and aleurone layer that are necessary for the seeds to germinate and develop into a new plant. A hallmark of the senescence process is the dismantling of the photosynthetic apparatus of the leaf chloroplasts (Krupinska *et al.*, 2013), by which the plastidic proteins are degraded and turned into compounds amenable for transport from the leaf to other organs of the plants, eventually to the grain during the maturation stage.

The genetic regulation of the complex senescence process is executed via the spatial and temporary patterns of gene expression during the developmental process of the plant. Hence, the temporal regulation of senescence-associated genes (SAGs) has been intensively studied over the last two decades (e.g. Lohman *et al.*, 1994). At the regulatory level, the changes in these gene expression patterns are governed by a network of gene transcription factors, which again, with respect to senescence, are regulated by a combination of time (i.e. ageing) and environmental cues. Presumably, the latter take place via the combined effects of plant hormones that channel the impacts from the environment into changes in gene expression patterns (Schippers *et al.*, 2007). There are strong indications that members of the NAC transcription factor family are particularly involved in the signalling pathways that regulate senescence (Breeze *et al.*, 2011). A number of studies on specific NAC genes show that several members of this transcription factor family also have impacts on the regulation and execution of the senescence process in cereals. Uauy *et al.* (2006) showed that accelerated senescence in wheat caused by the introgression of a trait for high grain protein content was in fact due to the presence of a NAC transcription factor gene, and a recent report (Zhou *et al.*, 2013) demonstrated the regulation of senescence in rice by *OsNAP/ONAC058*. In addition, several individual NAC genes in *Arabidopsis* have been shown to regulate senescence, for example *AtNAP/ANAC029* (Guo *et al.*, 2006), *ORE1/ANAC092* (Kim *et al.*, 2009), *ORS1/ANAC059* (Balazadeh *et al.*, 2011), *JUB1/ANAC042* (Wu *et al.*, 2012), and *NTL4/ANAC053* (Lee *et al.*, 2012).

In the present work, the changes in gene expression taking place during the senescence process in barley (*Hordeum vulgare*) were studied in order to substantiate the understanding of the senescence process in a typical cereal plant. It is demonstrated that the generic senescence programme, studied mainly in *Arabidopsis* (e.g. Breeze *et al.*, 2011), in terms of patterns of gene expression, is also operating in the barley crop plant. Selected results from a microarray experiment were confirmed by quantitative reverse transcription–PCR (qRT-PCR) and details on the co-expression of members of the NAC transcription factor gene family with a number of SAGs are shown. With the aim to elucidate further the importance of NAC transcription factors in the regulatory networks governing the senescence process, biologically relevant palindromic NAC binding sites (NACBSs) were searched for in the promoter regions of a large number of genes. A significant enrichment of NACBSs was found in the promoters of the differentially up-regulated genes during senescence. Together, these findings strongly support the notion of NAC transcription factors being involved in the regulation of the senescence process and, hence, to be important for regulation of the maturation processes taking place in cereal crop plants.

## Material and methods

### Plant material

For the microarray experiment, barley plants (*H. vulgare*, cv. Golden Promise) were grown in the greenhouse in pots containing a 50:50 peat–perlite mixture. A standard nutrient solution was supplied via the irrigation system. Artificial illumination was used for supplementation of sunlight and for ensuring a day/night cycle of 16/8h. Sampling of leaves was done between 12:00h and 14:00h. Non-senescing flag leaves were harvested ~5 d before anthesis, medium-senescing leaves at ~15 d post-anthesis, and senescing leaves at ~30 d post-anthesis, selecting leaves with ~50% green leaf area. Frozen homogenized leaf samples were used for RNA isolation as described previously (Christiansen *et al.*, 2011)

For the qRT–PCR experiments, barley plants, cv. Golden Promise, were grown in greenhouse soil plots with weekly sowings. Artificial illumination was used for supplementation and for ensuring a day/night cycle of 16/8h. Single flag leaves were harvested across the soil plot at different developmental stages, ranging from young leaves at the pre-anthesis stage to senescing leaves close to the grain maturity stage where the leaves were completely chlorotic, but still turgid. Frozen homogenized leaf samples were used for RNA isolation as described previously (Christiansen *et al.*, 2011) and for determination of total chlorophyll content, following extraction in 96% ethanol and UV measurements according to Lichtenthaler (1987). In the analyses of gene expression patterns, the single flag leaf samples were ordered according to decreasing chlorophyll content.

### Microarray experiment

The Agilent 4×44 Barley Gene Expression Microarray (Agilent, http://www.genomics.agilent.com, last accessed 12 February 2014) was used for the microarray experiments. Sample quality control, sample labelling, hybridization, scanning, and data extraction were performed by imaGenes (Berlin, Germany), according to the complete integrated Agilent Workflow Solution certified by Agilent (www.imagenes-bio.de, last accessed 12 February 2014). In brief, the isolated RNA was reversed transcribed into cDNA, followed by an *in vitro* transcription reaction during which Cy3 labels were incorporated into cRNA, which was subsequently hybridized to the microarray chip in single channel hybridizations. Data acquisition was performed with an Agilent DNA microarray scanner with Feature Extraction Software. The quality filtering and statistical analysis of the raw data obtained was performed with the Agi4×44PreProcess (Lopez-Romero, 2011) and Limma R-packages (Smyth, 2005).

The Agilent barley microarray used contained redundant sequences at two levels. (i) Several target sequences, defined by the same primary accession number, were represented by from two to several probes in the chip. (ii) Since many of the target sequences were partial expressed sequence tags (ESTs), several of them in fact represent the same gene sequence. To cope with the redundancy, only one probe for each target sequence was used in the analysis, selected via the following procedure: the probe with the highest or second highest signal closest to the 3′ end of the target sequence was selected as a representative probe. The distal 3′ probes were favoured since the labelling of cRNA for the chip hybridization was performed from the poly(A) tail. Furthermore, the redundancy was alleviated by making blastN searches of the Agilent Chip probes against recently released full-length cDNA sequences of barley (Sato *et al.*, 2009; Matsumoto *et al.*, 2011). Up to three mismatches to the 60 nucleotide long probes were allowed. Subsequently, redundant probes representing the same sequence were removed as described above.

Annotation of differentially expressed genes during senescence was done using the information for the Agilent 4×44 Barley chip available at http://mapman.gabipd.org (last accessed 12 February 2014), which also formed the basis for the MapMan categorization of the target genes into functional bins, supplemented by manual annotation of a limited number of genes. The MapMan bins (Usadel *et al.*, 2009) were used to analyse and visualize selected aspects of the differential gene expression during senescence.

### qRT–PCR experiments

Procedures for RNA isolation, cDNA synthesis, primer design, primer efficiency testing, and quantitative real-time PCR were performed as described previously (Christiansen *et al.*, 2011). Primers for 48 NAC genes were the same as used by Christiansen *et al.* (2011). Primers for qRT–PCR representing other genes in this study are listed in Supplementary Table S6 available at *JXB* online. Ct values exported from the ABI Prism 7900HT SDS software were used as raw data for the analysis of qRT–PCR data. The R software (R Development Core Team, 2005) and the add-on packages HTqPCR (Dvinge *et al.*, 2009) and Limma (Smyth, 2005) were used for the manipulation and analysis of the raw Ct values. qRT–PCR runs showing high variation among the three technical replicates were manually inspected, and clear outliers and runs with aberrant dissociation curves were excluded from the analysis. Several possible reference genes [*18S rRNA* (AK251731), *actin* (AY145451), *α-tubulin* (X99623), *EF1-α* (Z50789), *G6PDH* (AM398980), *HP68 protein* (AK251800), *HSP70* (AK248694), and *Splicing factor 2* (AK249101) (Faccioli *et al.*, 2007; Gregersen and Holm, 2007)] were tested for their stability across the range of tested samples using the tools in the R package SLqPCR (Kohl, 2007). Based on this analysis, the barley *18S rRNA* gene was selected as the most stable reference gene to be used in the normalization of gene expression. The *Splicing factor 2* (*SP2*) gene also showed high stability and was included in the presentation of the data as a control gene.

### *In silico* co-expression analysis

Genes in barley co-expressed with barley NAC genes were investigated *in silico* using the resources from http://coexpression.psc.riken.jp/barley/search.pl, last accessed 12 February 2014 (Mochida *et al.* 2011). Only genes included in the Affymetrix Barley1 Chip are available in these resources, and hence only 33 out of the 48 NAC genes from Christiansen *et al.* (2011) could be analysed. The rankings in the co-expression list for each of the 33 NAC genes were compared with the gene expression data from the senescence microarray and qRT–PCR results, in order to substantiate the co-regulations observed there.

### Promoter analysis

Putative barley promoter sequences (1000 bases upstream of ATG) and their corresponding coding sequences (CDS) were obtained from http://plants.ensembl.org/Hordeum_vulgare, last accessed 12 February 2014 (International Barley Genome Sequencing Consortium, 2012). For detection of putative NACBSs in the promoter sequences, the FIMO tool available at http://meme.nbcr.net/meme/cgi-bin/fimo.cgi (last accessed 12 February 2014) was used (Grant *et al.*, 2011).

## Results

### Agilent 4×44K barley microarray experiment on flag leaf senescence

In order to study the general changes in gene expression taking place during senescence of the barley flag leaf, a microarray experiment was performed on three stages of senescence of the flag leaf of greenhouse-grown barley plants (cv. Golden promise). Sampling was done from young, just fully developed barley flag leaves (~5 d before anthesis); from medium-senescing flag leaves (~15 d post-anthesis); and from strongly senescing flag leaves (green leaf area ~50%; ~30 d post-anthesis). Three samples, each comprising 3–5 leaves, were used for the first and last harvest time points, and two samples for the medium stage. This provided RNA samples for, in total, eight hybridizations distributed over two Agilent 4×44 Barley Microarray chips. The hybridizations were performed by imaGenes (Berlin, Germany), from whom the extracted raw data were received. These raw data are available from ArrayExpress (http://www.ebi.ac.uk/arrayexpress/, last accessed 12 February 2014; accession no. E-MTAB-2133). The data were analysed using the Agi4×44PreProcess and Limma R-packages. Herein, mainly the data from the contrast between non-senescing and strongly senescing samples will be presented and discussed. In a standard hierarchical clustering analysis of the samples, one of the senescing samples, Sen3, appeared as an outlier with considerably lower signal values than the other two senescence samples. Accordingly, the Sen3 sample was omitted in the final data analysis, which, however, only marginally affected the ordering of differentially expressed genes.

During pre-processing of the data in the Agi4×44PreProcess software, background correction was performed with the method ‘half’, and normalization across the eight hybridizations with the method ‘quantile’. Low quality features, in particular those with signals close to or below background levels, were filtered out, according to default settings of the Agi4×44PreProcess software. The pre-processing resulted in 24 646 features with acceptable signal values. The statistical analysis of these filtered data using the Limma software resulted in ordered lists of gene probes according to the probability of their differential expression across the different contrasts in the experiment (Supplementary Table S1 available at *JXB* online). According to the adjusted *P*-values of the Limma tests for differential expression, a large number of genes appeared as differentially expressed in the contrast between strongly senescing and non-senescing flag leaves, presumably reflecting the immense physiological changes taking place in the leaves during senescence. Since a decision on a precise threshold between significant and non-significant differential gene expression in studies like this is difficult (Smyth *et al.*, 2003), the top 5000 ranked probes (~20% of the filtered probes) were arbitrarily chosen for further analysis. The cut-off was at an adjusted *P*-value of ~0.00035 (see Supplementary Table S1 available at *JXB* online). The Agilent barley chip contained a high number of redundant sequences, which were handled according to the approach described in the Materials and methods. This resulted in a list of 3867 non-redundant differentially expressed genes that were subject to detailed analysis.

The MapMan tool was used to analyse the functional structure of the selected differentially expressed genes. This tool attempts to classify genes into functional bins at different levels of detail and allows versatile visualization of the relative expression levels of selected genes (Usadel *et al.*, 2009). The top-bins describe rather broad groups of gene functions, such as protein or lipid metabolism. Supplementary Fig. S1 and Table S2 available at *JXB* online show the distribution of the 3867 selected probes across the top-bins, illustrating that there were many both up- and down-regulated genes in senescing leaves in most categories. The exceptions were, in particular, categories involved in photosynthesis [bins for photosystems (1) and tetrapyrrole biosynthesis (19)], which showed predominantly down-regulation of genes. A number of the up-regulated genes in the photosynthesis category are related to the photorespiration process, and their encoded functions in the context of senescence might be related to the turnover of glycine also originating from sources other than the photorespiration cycle (Bauwe *et al.*, 2003). Top-bin 17, hormone metabolism, and top-bin 26, with miscellaneous functions, both showed a clear bias towards more up-regulated than down-regulated genes at this general functional level.

**Fig. 1.**
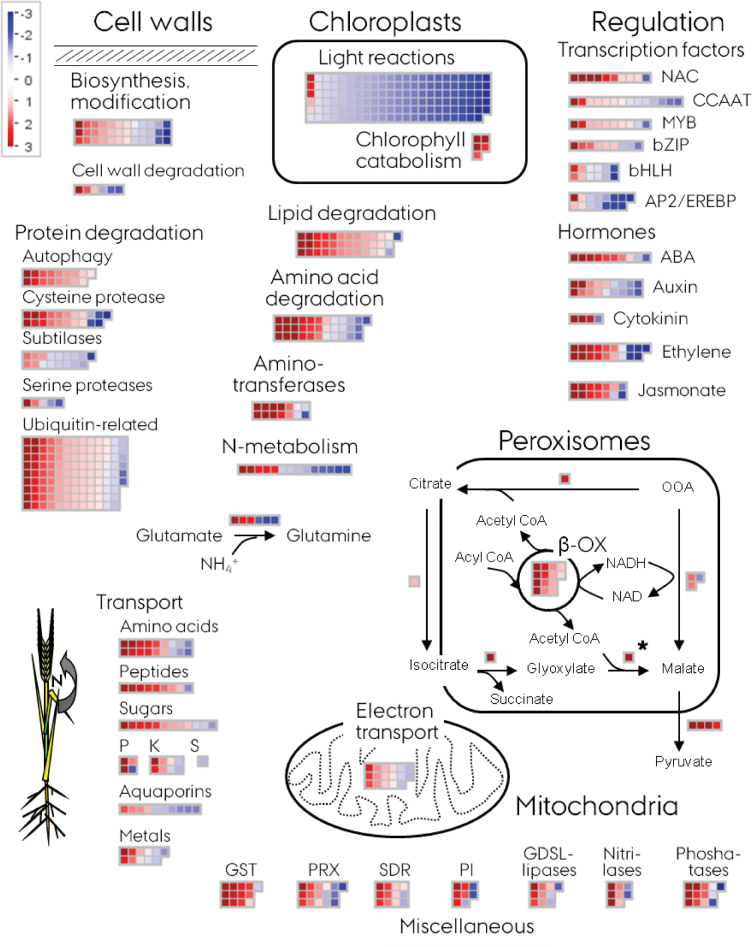
MapMan ‘senescence pathway’: the distribution of gene expression in senescing compared with non-senescing barley flag leaves across selected MapMan sub-bins, with function suggested to be involved in senescence processes. Blue indicates down-regulation, and red up-regulation of gene expression. Details on the individual genes are provided in Supplementary Table S2 available at *JXB* online. *The malate synthase gene expression is from a qRT–PCR experiment, since this gene (accession no. AK357164) was not represented in the Agilent chip.

In order to study in more detail the gene expression during senescence in barley, a number of subcategories were selected from the MapMan annotation that, based on the senescence literature, are believed to be involved in senescence-associated processes. To visualize this analysis, the ‘senescence pathway’ map in Fig. 1 was designed, using the MapMan tools for generating novel maps. Figure 1 shows the up-regulation of a large number of genes in the selected categories. The accession numbers and relative expression values for the different categories are listed in Supplementary Table S3 available at *JXB* online.

The senescence process, visually observed as yellowing of the leaves at the late sampling time point, was clearly reflected in the down-regulation of genes involved in the photosynthesis reactions taking place in the chloroplasts. In parallel, there was an up-regulation in the chloroplast of five genes involved in chlorophyll breakdown. Several different subcategories involved in protein and lipid degradation and carboxylic acid metabolism showed clear up-regulation. For protein degradation, the autophagy- and ubiquitin-related pathways, along with the cysteine peptidases, appeared as the predominantly up-regulated categories. The up-regulation of the lipid degradation pathways in the peroxisomes during senescence is well known (Yang *et al.*, 2009), supported here by the strong up-regulation of a number of genes involved in the β-oxidation of fatty acids taking place in peroxisomes. This was accompanied by an up-regulation of genes encoding enzymes usually described to be involved in the glyoxylate cycle: citrate synthase, aconitase, and isocitrate lyase. There was unfortunately no probe in the chip representing the malate synthase step of the glyoxylate cycle. However, supplementary qRT–PCR results showed that this gene (accession no. AK357164) was strongly up-regulated (log ratio 9.8) during leaf senescence in barley. This value is inserted in Fig. 1. The strong up-regulation of genes leading to formation of malate was also accompanied by strong up-regulation of a number of genes encoding malic enzymes.

Up-regulated genes associated with mitochondrial functions were mainly related to the electron transport chain, indicating up-regulation of respiratory processes via, for example, the alternative oxidase. There was no clear indication, from top-bin 8 for tricarboxylic acid (TCA) metabolism, of a general up-regulation of the TCA cycle of the mitochondria.

In accordance with the presumed translocation of compounds and mineral nutrients from senescing leaves, a number of transporter gene transcripts were up-regulated during senescence, mainly comprising transporters for amino acids, peptides, and sugars, but there was also a clear indication for up-regulation of transporters involved in transport of minerals.

Top-bin 26, with miscellaneous functions, which showed an overall bias towards up-regulated genes in senescing leaves (Supplementary Fig. S1 available at *JXB* online), was analysed for sub-bins that in particular represented this up-regulation, and the seven sub-bins shown in Fig. 1 were pinpointed. They appeared to represent categories of functions involved in redox and catabolic processes, such as peroxidases and phosphatases.

With respect to regulation of the senescence process, several genes encoding transcription factors or genes involved in hormone regulation were up-regulated in senescing leaves. For the hormones, several genes involved in the biosynthesis, degradation, or responses to hormones were up-regulated, supporting the notion that hormones play a role in the regulation of senescence (Schippers *et al.*, 2007). Regarding transcription factors, the NAC transcription factor family in particular had several members that were up-regulated during senescence. However, members of other transcription factor families were also transcriptionally up-regulated: bZIP, MYB, bHLH, AP2/EREBP, and CCAAT transcription factors.

With the aim to study further the association of NAC transcription factors with the senescence process, a more detailed analyses of the expression levels of these genes was carried out. Table 1 shows the comparison of the microarray results with the qRT–PCR results from Christiansen *et al.* (2011) whose gene expression experiments for the NAC genes were performed on similar, but not identical, senescing barley leaf material to that in the present study. Of the 48 barley NAC gene sequences described by Christiansen *et al.* (2011), only 27 were represented by probes in the Agilent chip. Seven of these probes had low expression levels that caused them to be filtered out during the pre-processing of the microarray data. Of the remaining 20 probes, 10 were among the selected differentially expressed genes at the late senescing time point as illustrated in Fig. 1. Only one, representing the *HvNAC046* gene, showed down-regulated expression in senescing leaves. To illustrate the reproducibility of the results from the Agilent chip by qRT–PCR, Supplementary Fig. S2A available at *JXB* online shows the strong correlation between the senescence-associated expression levels of the barley NAC genes from the two experimental systems. There was a clear overlap between the NAC genes that showed significant differential expression in the two experimental approaches.

**Table 1.** Barley NAC genes used in this study, with GenBank accession numbers, corresponding names of contigs in the Barley1 chip, and probe names for the Agilent microarrayGene expression fold changes (FC, log2 scale) in the microarray experiments are given for leaves at intermediate (M15D) and late (SEN) senescence stages (NS, non-senescing flag leaf). The qPCR data give the FC of expression in senescing compared with non-senescing flag leaves from Christiansen *et al*. (2012). Mean intensity and mean Ct values indicate the relative gene expression levels in the microarray and the qPCR experiments, respectively.

Name	Accession no.	Barley1 chip	Agilent microarray				qPCR	
				Log2 FC				
		Contig name	Probe_name	M15D versus NS	SEN versus NS	Mean intensity (log2)	Log2 FC	Mean Ct
HvNAC001	AK250475	HD05L07r_at	A_13_p078941	2.27	**4.05**	9.01	3.73	22.62
HvNAC002	AK249396	Contig5723_at	A_13_p017131	0.08	0.20	11.46	–1.12	17.58
HvNAC003	AK249102	Contig3361_at	A_13_p421415	0.61	**1.47**	13.52	2.78	16.84
HvNAC004	AM500853	Contig3362_at					1.57	19.64
HvNAC005	AK251058	Contig14026_at	A_13_p016456	2.47	**3.95**	9.07	5.39	21.29
HvNAC006	AM500854	Contig6233_at					3.30	17.46
HvNAC007	AK249749	Contig10340_at	A_13_p015456	0.36	**0.94**	12.16	1.29	19.98
HvNAC008	FR821737	Contig9031_at	A_13_p150635	0.80	**2.05**	13.42	3.36	19.56
HvNAC009	FR819761	Contig17688_at					–0.34	15.79
HvNAC010	FR821754	Contig13345_at	A_13_P093105	1.31	**2.25**	13.86	2.32	17.62
HvNAC011	AK251493	HVSMEa0011M12r2_at					4.02	23.5
HvNAC012	FR819762						–3.20	21.89
HvNAC013	AK376297	Contig11098_at	A_13_P553194	1.27	**4.43**	10.93	5.19	18.54
HvNAC014	FR821738	Contig9757_at					2.81	22.25
HvNAC015	FR821739	Contig6484_at	A_13_P068461	0.03	0.06	6.67	–0.21	21.20
HvNAC016	AK366470	Contig5241_at	A_13_p136620	0.21	**0.98**	12.61	1.54	15.57
HvNAC017	FR821740	Contig8993_at	A_13_p576969	^*a*^	^*a*^		4.04	22.73
HvNAC018	FR821741	Contig9284_at					3.97	23.47
HvNAC019	FR819764						3.67	21.99
HvNAC020	FR821742	Contig10172_at	A_13_p094660	0.03	0.56	5.90	2.94	19.56
HvNAC021	AK370287	Contig15867_at	A_13_p002701	^*a*^	^*a*^		3.92	22.61
HvNAC022	AK365398						2.72	21.72
HvNAC023	FR821745	Contig13658_at					2.25	22.58
HvNAC024	FR821746	Contig11340_at	A_13_p341882	^*a*^	^*a*^		3.81	22.80
HvNAC025	AK364002		A_13_P180884	–0.01	0.76	5.95	3.73	22.97
HvNAC026	FR819767		A_13_P554129	^*a*^	^*a*^		2.94	21.43
HvNAC027	AK368213	HM07L17r_at	A_13_P042621	2.39	**5.29**	8.36	6.43	19.90
HvNAC028	AB362161	HVSMEk0006G11r2_s_at	A_13_p085361	0.07	0.16	5.94	0.38	17.74
HvNAC029	EU908210						3.29	19.79
HvNAC030	DQ869679						4.24	24.64
HvNAC031	AY672069		A_13_p080271	–0.43	–0.46	9.26	–0.53	18.75
HvNAC032	AK248480		A_13_p100800	^*a*^	^*a*^		3.42	23.81
HvNAC033	AK248449	Contig19673_at					3.10	25.66
HvNAC034	AK249120		A_13_p100355	–0.27	0.13	7.62	2.00	22.56
HvNAC035	FR821748	Contig5740_at	A_13_p098010	–0.09	0.12	9.06	1.37	18.31
HvNAC036	AL505464						4.18	21.53
HvNAC037	AK371156						6.06	19.51
HvNAC038	BY847894						2.74	22.04
HvNAC039	AK370035	Contig11856_at					3.62	21.88
HvNAC040	AK361879	Contig15251_at					2.36	20.96
HvNAC041	FR821751	Contig7432_at	A_13_P133280	0.36	0.85	12.72	–0.76	16.84
HvNAC042	AK361273	Contig17088_at	A_13_P069701	^*a*^	^*a*^		–1.58	20.63
HvNAC043	GH216054						2.12	22.81
HvNAC044	AK364683	Contig19862_at					1.24	20.85
HvNAC045	BF259201		A_13_P292932	^*a*^	^*a*^		2.96	24.4
HvNAC046	AK252960	Contig12579_at	A_13_P246531	–1.19	**–1.74**	8.88	–2.01	18.59
HvNAC048	AK355552	Contig13898_at	A_13_P157980	0.16	–0.03	11.29	0.82	18.97

^*a*^ Probes were filtered out during pre-processing of microarray data, due to low intensity levels.

FC values in bold indicates NAC genes that are among the genes selected as differentially expressed in senescing leaves.

### qRT–PCR experiments

**Fig. 2.**
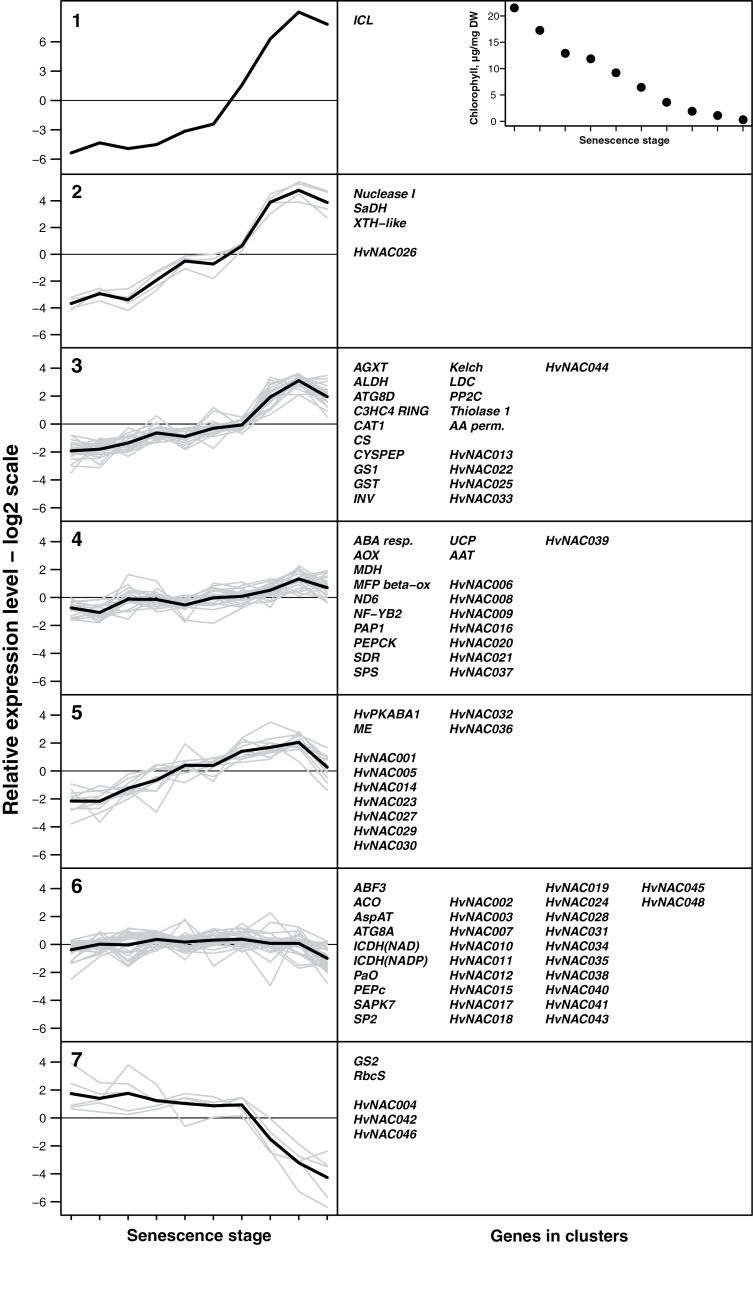
K-means clustering of gene expression patterns for 44 selected senescence-associated genes and 47 NAC genes, as measured by relative qRT–PCR across 10 samples of barley flag leaves at progressive stages of senescence. The samples were ordered according to the chlorophyll content, shown in the insert at the upper right corner. Names of genes in the individual clusters are given in the right column of boxes. Abbreviations of gene names are shown in Table 2.

In order to study in more detail the regulation patterns of SAGs and the NAC genes during the progress of senescence, 44 SAGs were selected for detailed qRT–PCR studies across the different functional categories shown in the MapMan ‘senescence pathway’ in Fig. 1. Primarily genes encoding well-described functions were selected. The list of selected SAGs is given in Table 2 along with their expression levels in the microarray experiment. As input material for the qRT–PCR experiment, single barley flag leaves were harvested from plants at different stages of development from the pre-anthesis stage to completely senescing leaves. The chlorophyll content was determined and used as a measure of senescence progress, and expression levels for the selected genes and 47 barley NAC genes (Christiansen *et al.*, 2011) were determined by qRT–PCR. The *18S RNA*-normalized gene expression patterns were clustered by K-means clustering as implemented in the R software (R Development Core Team, 2005). Following a number of test clusterings and supported by a rule of thumb of √(*n*/2) clusters for k-means clustering, seven clusters were chosen as an optimal cluster number that divided the genes into distinct groups. Figure 2 shows the resulting clusters for 10 samples across the span of chlorophyll contents. The strong correlation between results from the Agilent chip and the qRT–PCR experiment for the selected SAGs are shown in Supplementary Fig. S2B available at *JXB* online. The difference in expression level between sample 9 and 1 from the qRT–PCR experiment was used as the senescence-associated expression that could be compared with the microarray expression results.

**Table 2.** Barley senescence-associated genes used for the qRT–PCR experiment in Fig. 2, with probe names for the Agilent microarray, acession numbers (GenBank or http://plantta.jcvi.org/, last accessed 12 February 2014), blastX hits, abbreviations used in Fig. 2, and expression data from the microarray experimentThe genes were selected across different categories of gene functions shown in Fig. 1.

Category	Probe name	Accession number	Putative function (BlastX hit)	Abbreviation in Fig. 2	Agilent chip		
					Fold change (log2)	Average expression level	Adjusted *P*-value
Carboxylic acid metabolism							
	A_13_P479945	AK354442	Aconitate hydratase, cytoplasmic	ACO	0.99	11.4	5.55E-05
	A_13_P529474	AK361069	Alanine-glyoxylate aminotransferase	AGXT	4.50	9.6	0.000103
	A_13_P114535	AK364160	Aspartate aminotransferase	AspAT	1.92	10.2	9.14E-05
	A_13_P518849	AK252959	Citrate synthase, peroxisomal	CS	2.82	13.4	6.47E-06
	A_13_P008951	AK252731	Glutamate decarboxylase	GAD	0.84	13.9	0.000145
	A_13_P113710	TA28480_4513	Glutamine synthetase 1	GS1	2.94	13.2	1.67E-05
	A_13_P125240	AK364289	Glutamine synthetase 2	GS2	–2.88	10.2	2.51E-05
	A_13_P014696	AK252018	Isocitrate lyase	ICL	11.60	8.2	2.40E-06
	A_13_P135625	AK368534	Malate dehydrogenase, peroxisomal	MDH	1.84	8.3	2.09E-05
	A_13_P130815	AK360118	NAD-dependent isocitrate dehydrogenase	ICDH (NAD)	0.85	11.8	0.001031
	A_13_P134780	TA34549_4513	NADP malic enzyme, cytosolic	ME-2	5.92	8.1	2.51E-05
	A_13_P094210	AK252839	NADP-dependent isocitrate dehydrogenase	ICDH (NADP)	1.00	14.1	0.002635
	A_13_P037031	AK250093	NADP-dependent malic enzyme	ME-1	3.65	12.9	3.48E-06
	A_13_P140540	TA36323_4513	Phosphoenolpyruvate carboxykinase	PEPCK	3.46	8.0	1.67E-05
	A_13_P242685	TA35051_4513	Phosphoenolpyruvate carboxylase	PEPc	–1.33	9.5	0.000139
Fatty acid metabolism							
	A_13_P355167	BG418084	3-Ketoacyl-CoA thiolase 2, peroxisomal	Thiolase 1	3.14	12.9	3.30E-06
	A_13_P008556	AK251077	MFP glyoxysomal fatty acid beta-oxidation	MFP beta-ox	1.91	13.7	1.23E-05
	A_13_P540524	AK372098	Short-chain dehydrogenase/reductase (SDR)	SDR	1.88	11.1	1.56E-05
Carbohydrate metabolism							
	A_13_P067481	AK365330	Endotransglucosylase/hydrolase	XTH-like	9.62	11.1	6.47E-06
	A_13_P583324	AK367512	Extracellular invertase	INV	3.23	14.1	5.09E-05
	A_13_P157610	TA41920_4513	Sucrose-phosphate synthase	SPS	2.55	10.4	1.55E-05
Protein metabolism							
	A_13_P177459	AK376180	Lysine decarboxylase-like	LDC	5.11	11.2	6.75E-06
	A_13_P157210	AK358908	Papain-like cysteine peptidase	CYSPEP	3.31	11.3	5.68E-06
	A_13_P142895	TA37051_4513	Saccharopin dehydrogenase-like	SaDH	8.46	9.9	2.90E-07
Autophagy							
	A_13_P361137	BI947475	Autophagy-related protein 8A	ATG8A	1.97	14.6	5.30E-05
	A_13_P515061	AK250515	Autophagy-related protein 8D	ATG8D	3.70	10.7	4.99E-07
Nucleic acid metabolism							
	A_13_P000061	AB028448	Nuclease I	Nuclease I	8.06	11.2	3.90E-06
Kinases/phosphatases							
	A_13_P077736	AK372880	Protein kinase HvPKABA1	HvPKABA1	6.01	9.9	1.07E-05
	A_13_P062196	AK356066	Protein phosphatase 2C	PP2C	3.41	9.6	2.33E-05
	A_13_P144685	AK250358	Serine/threonine-protein kinase SAPK7	SAPK7	1.82	10.5	8.88E-06
Electron transport chain							
	A_13_P085996	AK363239	Alternative oxidase	AOX	2.33	12.1	0.00016
	A_13_P011006	AK251682	Mitochondrial uncoupling protein	UCP	1.87	10.0	2.16E-05
	A_13_P189899	TA53161_4513	NADH-ubiquinone oxidoreductase chain 6	ND6	3.80	7.9	0.000152
	A_13_P470383	AK376855	Succinate dehydrogenase	SuDH-2	2.06	9.2	2.24E-05
Miscellaneous							
	A_13_P008261	AK370899	ABA responsive element binding factor 3	ABF3	-0.16	10.1	0.265021
	A_13_P421145	AK363564	Aba-responsive protein	ABA resp.	3.60	12.3	5.04E-06
	A_13_P002226	AK252220	Aldehyde dehydrogenase, cytosolic	ALDH	4.07	11.8	1.80E-06
	A_13_P460818	AK353635	Amino acid permease	AA perm.	5.53	9.7	2.40E-06
	A_13_P137455	AK361903	Amino acid transporter	AAT	4.42	7.5	4.52E-06
	A_13_P542217	DN186744	Catalase 1	CAT1	3.57	7.1	1.26E-05
	A_13_P234439	AK357085	Ferritin	Ferritin	3.71	15.1	2.40E-05
	A_13_P135220	AK371360	Glutathione transferase	GST	2.59	11.4	3.66E-05
	A_13_P139245	TA35901_4513	Kelch repeat-containing F-box-like	FBK	4.17	10.3	4.99E-07
	A_13_P278734	AK353593	Pheophorbide a oxygenase, chloroplastic	PaO	2.25	13.4	1.28E-05
	A_13_P132770	AK375026	Purple acid phosphatase 1	PAP1	1.69	12.1	2.45E-05
	A_13_P081911	AK252711	RUBISCO small subunit	RUBISCO	–2.53	14.9	0.000144
	A_13_P399036	AK249101	Splicing factor 2	SP2	–0.29	6.2	0.289038
	A_13_P153380	AK370727	Transcription factor subunit NF-YB2	TF NF-YB2	2.05	10.2	1.08E-05
	A_13_P171920	AK368972	Zinc finger (C3HC4-type RING finger)-like protein	C3HC4 RING	3.91	10.4	2.62E-06

Since mostly up-regulated SAGs were selected, most of the clusters in Fig. 2 also showed up-regulation of gene expression, or only slight changes in expression, during the progress of senescence; however, with distinct patterns for the different clusters. Cluster 7 was the only cluster that showed clear down-regulation at the later stages of senescence. Genes encoding Rubisco small subunit and glutamate synthase 2 were selected as known markers of senescence, and three NAC genes showed a similar down-regulation to that of these marker genes. Cluster 6 comprised a large group of genes, amongst them almost half of the 47 NAC genes, which seemed only marginally affected by the progress of senescence. Accordingly, the apparent up-regulation of many of these genes in the microarray experiment, for example *PaO*, *ACO*, or *ATG8A*, was not reflected in the expression profile of this cluster, apart from a slight transient up-regulation at the mid-stages. The NAC genes *HvNAC003*, *-7*, and *-10* were included in cluster 6, and the apparent up-regulation in the microarray of these three NAC genes was hence not confirmed.

Clusters 1–5 all showed up-regulation of gene expression towards the later stages of senescence, however, with different patterns. Cluster 1 showed the extreme up-regulation of an isocitrate lyase gene (*ICL*), which confirmed the strong up-regulation also observed in the microarray experiment. None of the other selected SAGs had a similar expression pattern and hence the *ICL* gene was the only gene in cluster 1. In the microarray experiment, the *ICL* probe represented by far the most strongly up-regulated gene on the chip, with a log2-fold change of 11.6. Cluster 2 also showed strong, ~8-fold (log2) up-regulation of three SAGs, encoding nuclease I, saccharopine dehydrogenase, and a xyloglucan *endo*-transglycosylases/hydrolase (*XTH*). Again, this confirmed the strong up-regulation also observed in the microarray data of these genes. Cluster 2 also showed the strong up-regulation of one NAC gene, *HvNAC026*, encoding a close homologue of *AtXND1*, a putative negative regulator of xylem development in *A. thaliana* (Zhao *et al.*, 2008). This gene was represented in the Agilent chip, but due to very low signal levels in the non-senescing samples, it was filtered out during the pre-processing of the microarray data. In the qRT–PCR experiment, the expression level in non-senescing tissue was very low as well, which then gave rise to a strong relative up-regulation at the later stages of senescence.

Clusters 3, 4, and 5 all showed a clear increase in transcript levels over the developmental panel of flag leaves, however, with some noticeable differences. Cluster 3 showed a relatively strong peak towards the end of senescence development. Compared with that, cluster 5 showed an increase in expression levels at earlier senescence stages. Cluster 4 showed an intermediate pattern between cluster 3 and 5. Cluster 3 contained a number of genes encoding products involved in turnover of proteins, lipids, and carboxylic/amino acids, for example a papain-like cysteine peptidase, a thiolase, and a glutamine synthase. Four NAC genes were present in cluster 3, three of which (*HvNAC013*, *-22*, and *-25*) belonged to the NAC-d group (Christiansen *et al.*, 2011). Clusters 4 and 5 differed mainly in the amplitude of transcript accumulation. Whereas cluster 5 showed around a 4-fold (log2) difference between the early and late stages of senescence, cluster 4 only showed an ~2-fold (log2) difference. Cluster 5 also showed a tendency to level off towards the later stages of senescence. This cluster contained nine of the up-regulated NAC genes, and, noticeably, five of these (*HvNAC005*, *-23*, *-27*, *-29*, and *-30*) belong to the same phylogenetic group, the NAC-a group (Christiansen *et al.*, 2011).

### NACBSs in barley gene promoters

Several studies of the DNA binding activity of NAC domain proteins have determined a number of DNA sequence motifs (NACBSs) with high affinity for binding to NAC domains. They all contain a CGT core sequence with varying number of surrounding consensus nucleotides, which is thought to facilitate binding of the NAC transcription factors of promoter elements (see Table 3 for references). Hence, the core CGT sequence appears important for the DNA binding of many NAC transcription factors. However, with such a short motif, it is difficult to determine target genes based on promoter sequence analysis, and, when looking outside the core sequence, the consensus is not strong, making it difficult even with longer motifs to determine target genes. From the published consensus sequences, there are, however, in addition to the occurrence of the CGT core sequence, two other striking features: (i) the core sequence seems to occur in repeats, either direct or, most often, inverted repeats (palindromes), with varying number of spanning nucleotides; and (ii) in several cases the core sequence is preceded by T in the –2 and/or –3 positions. Preliminary data are available from studies on barley NAC domain proteins (HvNAC005 and -013; Søren Lindemose, personal communication), indicating that the consensus NACBSs in barley obey similar rules.

**Table 3.** DNA-binding motifs for NAC domain proteins with the CGT sequence as an essential core of the NAC-binding sequence (NACBS)

Reference	Plant species	Methodology	NAC protein	Suggested NACBS
Xie *et al.* (2002)	*A. thaliana*	Test of putative NACBS from 35S promoter	NAC1 (ANAC021)	CTG**ACG**TAAGGGATG**ACG**CAC
Olsen *et al.* (2005)	*A. thaliana*	CASTing to select binding oligonulceotides	ANAC019 ANAC092	[TA][TG][TAGC]**CGT**[GA] T[TAG][GA]**CGT**[GA][TCA][TAG]
Xue *et al.* (2006)	*T. aestivum*	CELD-fusion method	TaNAC69	GAGATC***CGT*** *G*CACAGT**ACG**TAACTGTTACA GA*GGTGTTT*AATGTTTA*C* ***ACG*** *TCTCT*AGTG
Zhang and Gan (2012)	*A. thaliana*	EMSA on *SAG113* promoter sequence	AtNAP (ANAC029)	C**ACG**TAAGT
Balazadeh *et al.* (2011)	*A. thaliana*	CELD-fusion method	ORS1 (ANAC059)	[AG]**CGT**[AG] (4–5n)[AG][CT]**ACG**CAA
Wu *et al.* (2012)	*A. thaliana*	CELD-fusion method	JUB1 (ANAC042)	TGC**CGT(**7n)**ACG** TGC**CGT(**7n)CCGC
Welner *et al.* (2012)	*A. thaliana*	DNase I footprinting		TTG**CGT**G(6n)C**ACG**CAA

In order to investigate the possible regulation of SAGs by NAC transcription factors in barley, seven model NACBS motifs (NACBS-1, -5, -6, -7, -8, -9, and -10; see Supplementary Table S4 available at *JXB* online for details) were designed based on the information in Table 3, with one repeat of the core sequence as a non-variable part, and with a 5–10 nucleotide span to an inverted repeat. Minor weights were given to less conserved nucleotides outside the core, based on the suggested NACBSs in Table 3. Only the NACBS-1 model did not have an inverted repeat, but instead had a strong conservation of T in the –2 and –3 positions relative to the CGT core. The model NACBS motifs were described in letter probability matrices and used in FIMO searches at http://meme.nbcr.net/meme/cgi-bin/fimo.cgi (last accessed 12 February 2014) against available promoter sequences of genes represented in the Agilent barley chip. The Agilent chip probe sequences were assigned to promoter sequences via blastN searches against the downloaded CDS sequences. This process presumably again reflected the sequence redundancy in the Agilent chip, inasmuch as only 12 643 unique promoter sequences were found for the 24 646 filtered probes from the analysis of the microarray experiment. However, the relatively low number might also reflect the provisional status of the gene models in the current barley genome sequences.

FIMO searches for the individual model motifs were performed on 2435 promoters found among the 3867 differentially up- or down-regulated genes, and on all 12 643 promoters. The results (Table 4) show that there was a significant over-representation of the model NACBSs in promoters from up-regulated genes, but not in promoters of down-regulated genes. A threshold level of *P*=1.0E-4 was used in the FIMO analysis (Grant *et al.*, 2011). There were a total of 532 hits across the eight NACBS models for the up-regulated genes, but since some of them targeted the same promoter sequence, 333 non-redundant promoter sequences contained at least one of the putative NACBS motifs.

**Table 4.** *Occurrence of putative binding sites for NAC transcription factors (NACBSs) in the promoters of senescence-associated barley genes*Promoter sequences (1000 bases upstream of ATG) were obtained from http://plants.ensembl.org/Hordeum_vulgare (last accessed 12 February 2014), and searched with the model NACBSs using the FIMO tool at http://meme.nbcr.net/meme/cgi-bin/fimo.cgi (last accessed 12 February 2014).

Name of NACBS	Model NACBS motif^*a*^	All genes (12 643)	Down-regulated genes (1329)	Up-regulated genes (1106)
NACBS-1	nttn**CGT**gnnn	817	84NS	108**
NACBS-5	ttn**CGT(**n)_5_ACGnnn	619	68 NS	80**
NACBS-6	ttn**CGT(**n)_6_ACGnnn	511	68*	56*
NACBS-7	ttn**CGT(**n)_7_ACGnnn	556	51NS	69**
NACBS-8	ttn**CGT(**n)_8_ACGnnn	747	74NS	93**
NACBS-9	ttn**CGT(**n)_9_ACGnnn	459	54NS	64**
NACBS-10	ttn**CGT(**n)_10_ACGnnn	529	73**	62**
	Total, non-redundant	2813	321NS	333**
	Per 1000 genes	222	242	301

Asterisks indicate statistical significance at the *P≤0.05 and**P≤0.01 level, using a hypergeometric test. NS, not significant.

^*a*^See Supplementary Table S4 available at *JXB* online for details on the weight for each nucleotide base.

### *In silico* co-expression studies

**Fig. 3.**
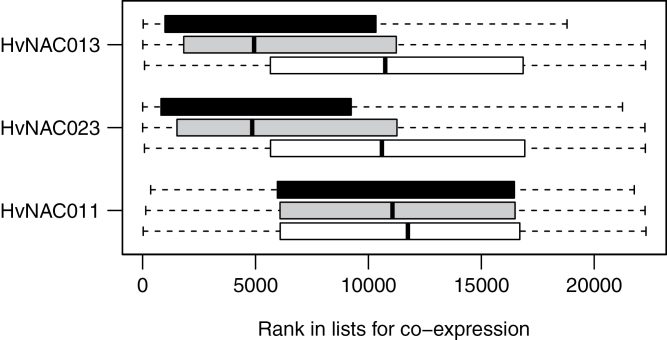
The distribution in co-expression lists for three NAC genes (*HvNAC013*, *-23*, and *-11*) of the 71 senescence-up-regulated genes having NACBS-8 in their promoter (dark grey), 1181 up-regulated genes without any NACBS (light grey), and 500 randomly selected genes (white). The lower the rank, the higher the co-expression with the indicated NAC genes (Mochida *et al.*, 2011).

In order to validate the results on putative NACBSs amongst the up-regulated genes during senescence, the overall gene co-expression with NAC genes was investigated in the barley co-expression database available at http://coexpression.psc.riken.jp/barley/search.pl, last accessed 12 February 2014 (Mochida *et al.*, 2011). Since this database is built on data from the Affymetrix Barley1 microarray chip, only genes represented by probes in this chip could be studied. The co-expression lists for the 32 NAC genes that were represented in the Barley1 chip (Table 1) were downloaded. The distribution of the up-regulated genes containing a putative NACBS in the lists ranked for co-expression with NAC genes were extracted. Indeed, the NACBS- containing genes overall showed tendencies of co-regulation with a number of the NAC genes, interpreted from a distribution in the co-expression lists biased towards the top (data not shown). Looking in more detail, it was evident that this distribution was most prominent for NACBS-8 with a span of eight nucleotides between the two inverted repeats of the core motif. The analysis was confined to the 71 up-regulated genes having the NACBS-8 motif in the promoter (see Supplementary Table S5 available at *JXB* online for details on these genes) and distributions in the co-expression lists for the 32 NAC genes were obtained as illustrated in Supplementary Fig. S3 available at *JXB* online. A number of NAC genes clearly showed a higher degree of general co-expression with the 71 genes having the NACBS-8 in the promoter than with up-regulated genes without any NACBS motif in the promoter. This was particularly prominent for *HvNAC008*, *-13*, *-23*, and *-44* that were all located in clusters in Fig. 2 with considerable up-regulation during senescence. As examples, distributions of genes with or without NACBS-8 in the co-regulation lists for *HvNAC013* and *-23* (both with high co-regulation) and for *HvNAC011* (low co-regulation) are shown in Fig. 3. It is not surprising that genes without any NACBS in the promoter also show tendencies of co-regulation with NAC genes, since indirect effects via regulatory networks and other regulatory pathways are also involved in the regulation of senescence. Furthermore, some of the NAC genes (e.g. *HvNAC013*) themselves contain NACBSs in their promoter, and may, hence, be part of gene activation cascades. In conclusion, the co-expression data support the notion that the occurrence of NAC-binding sites in the promoters of SAGs is of importance for the up-regulation of these genes during senescence in barley.

## Discussion

When the microarray experiment described in this work was conceived, the barley Agilent 4×44 Barley Microarray was the platform that had the best representation of the barley gene transcriptome. During the course of the experiment and the analysis of the results, new data sources for barley genomics information have become available, in particular the release of 24 000 full-length barley cDNA sequences (Sato *et al.*, 2009; Matsumoto *et al.*, 2011) and the recent release of a draft barley genome (International Barley Genome Sequencing Consortium, 2012), which provide valuable new information for genomics research in barley. An attempt was made to update relevant information for the Agilent microarray content according to these new data sources, for example in relation to redundancy in the probe representation of genes and in the analysis of promoter elements.

The rationale for carrying out an analysis of the senescence process in barley, considering that a number of comprehensive studies on senescence in *Arabidopsis* have been performed (Buchanan-Wollaston *et al.*, 2003,^2005^; Breeze *et al.* 2011) is that it is important to understand the senescence process in more detail in cereal plants, since this process is part of the maturation of cereal crop plants and hence of importance for the determination of crop yield and grain quality. Hence, this study adds to results from previous studies in cereals with more limited or different scopes (Gregersen and Holm., 2007; Jukanti *et al.*, 2008).

In the analysis of the microarray data, a MapMan ‘pathway’ map for the senescence process in barley was constructed, as shown in Fig. 1, in order to place a visual structure on top of the gene expression results. The map illustrates the coordinated up-regulation of degradation processes, along with transport and regulatory components. The categories of genes emerging from the analysis are in good alignment with previous gene expression analyses of the senescence process both in dicots (Breeze *et al.*, 2011) and in monocots (Gregersen and Holm, 2007; Jukanti *et al.*, 2008). This comprised categories with a large number of genes involved in protein degradation, in particular the categories of autophagy, cysteine proteases, and ubiquitin-related protein turnover. There was, however, only a partial overlap between the present results and those of Jukanti *et al.* (2008) when looking at the detailed probe level. Of the 3867 differentially regulated genes in the present study, only ~700 were found among the 2927 genes listed in the comparison between leaves of a stay-green barley cultivar and an early senescing cultivar in the work of Jukanti *et al.* (2008). This discrepancy presumably reflects different experimental set-ups, in addition to considerable noise in the analysis of microarray data for both studies.

The inter-related up-regulation of amino acid and lipid metabolism seems central to the senescence process, and here peroxisomal processes appear highly involved. In the end, this complex of up-regulated processes is believed to work as a way of rescuing nitrogen released during protein degradation for translocation in the form of amino acids to other plant parts, in particular to the developing grain (Feller *et al.*, 1994). In the peroxisome, a glyoxylate cycle-like process going from isocitrate to succinate and malate appears to be up-regulated, with the isocitrate lyase gene being the strongest up-regulated gene in the present study. The supplementary qRT–PCR experiment with malate synthase also showed the encoding gene to be strongly up-regulated. However, during senescence the glyoxylate pathway is probably not—as in a textbook description of it—connected to gluconeogenesis. More probably, it is part of anaplerotic pathways that, through the actions of the up-regulated amino acid transferases, could convert products from the fatty acid breakdown via malate/pyruvate into carbon skeletons (Brown *et al.*, 2010). These can be utilized in the re-assimilation of nitrogen via the glutamine synthetase reaction. This latter reaction stands as the central hub for the presumed translocation of nitrogen from senescing cereal leaves (Kichey *et al.*, 2005), and accordingly the two probes representing cytosolic glutamine synthetase (*GS1*) showed clear up-regulation. In contrast, three probes showed strong down-regulation of the chloroplastic *GS2* gene. There was no indication of up-regulation of the mitochondrial TCA cycle for the generation of NADP(H) reducing agents or for anaplerotic purposes. The possible needs for these agents for the cells were presumably met via the massive up-regulation of lipid and protein degradation processes. Mitochondrial functions that appeared to be up-regulated were mainly related to the electron transport chain, reflecting possible needs for ATP generation or for channelling of energy into dissipating processes, for example via the alternative oxidase.

At the regulatory level, the up-regulation of a number of genes encoding transcription factors was evident, involving genes encoding NAC, bZIP, MYB, bHLH, AP2/EREBP, and CCAAT transcription factors. In their comprehensive gene expression time course study, Breeze *et al.* (2011) showed these same families to contain the most strongly up-regulated transcription factor genes during senescence in *Arabidopsis*. Hence, there seems to be a conservation of the senescence process even at the complex regulatory level across the plant kingdom. The family of NAC transcription factors increasingly stands out as a key regulatory family for the senescence process and for abiotic stress responses (Nakashima *et al.*, 2012). The microarray data strongly supported that genes of this gene family in barley in particular are highly associated with the senescence process. This was previously indicated by studies with other species as well, in particular in *Arabidopsis* (Balazadeh *et al*., 2008; Breeze *et al.*, 2011), but also in wheat (Gregersen and Holm, 2007). Individual NAC genes have also been shown to regulate directly various parts of the senescence network (e.g. Guo *et al.*, 2006; Kim *et al.*, 2009; Balazadeh *et al.*, 2011; Wu *et al.*, 2012); however, the combined role of this gene family in regulating the complex senescence process has yet to be investigated in more detail. This is particularly pertinent since a high degree of redundancy in their role and functions might be expected with the occurrence of the high number of closely related genes in the genome.

In this study, the up-regulation of NAC genes during senescence was confined to a limited number of genes. Not all the NAC genes were represented in the microarray, and hence the qRT–PCR extended the number of studied barley NAC genes to 47 well-characterized genes (Christiansen *et al.*, 2011). The qRT–PCR experiment (and results from Christiansen *et al.*, 2011) confirmed the microarray results on differential regulation during senescence of seven out of 10 NAC genes. Furthermore, the qRT–PCR experiment (supported by unpublished data) showed up-regulation of an additional 8–10 NAC genes. Hence, in order to summarize the findings on NAC genes and regulation of senescence from this work, ~15 genes that are up-regulated during senescence can be listed, with some of the genes having strong and reproducible expression patterns whereas others are more variable across experiments. Based in particular on their different expression patterns, they can be divided into three groups: (1) *HvNAC026* stands out on its own as a very strongly up-regulated gene with almost no expression in non-senescing leaves. The most closely related gene in *Arabidopsis*, *AtXND1*, is not among the SAGs selected by Breeze *et al.* (2011); however, in the gene expression atlas presented by Schmid *et al.* (2005), *AtXND1* is clearly up-regulated in senescing *Arabidopsis* leaves. Nevertheless, the present report is the first one to place a focus on this gene as senescence associated. (2) A group of genes with a relatively early up-regulation that tend to level off towards the later stages of senescence, exemplified by the closely related NAC-a genes *HvNAC005*, *-23, -27*, *-29*, and *-30*. (3) Genes up-regulated during the late burst of degradation processes in the leaf tissues. This is exemplified by the NAC-d genes *HvNAC013*, *-22*, and *-25*. Further studies are needed in order to fine-tune this classification. However, a preliminary hypothesis would be that the group 2 genes, with their early induction, are involved in signalling processes, inasmuch as this group of genes seems to be associated with abscisic acid (ABA) processes. Most of them are up-regulated following ABA treatment (Christiansen *et al.*, 2011) and the closest homologue of *HvNAC005* and *-23* in *Arabidopsis* is the *AtNAP* gene, a known regulator of ABA signalling (Zhang *et al.*, 2012). The group 3 genes might be involved in regulation of the final execution of the degradation process by direct regulation of genes encoding degradation enzymes. Evidence supporting this is that one of the strongest co-regulated SAG genes with the group 3 NAC genes, the cysteine protease gene AK358908, does indeed have an NACBS in its promoter (data not shown). Exactly which NAC transcription factor binds to this site still needs experimental testing.

One of the ways to study the direct regulation of target genes of transcription factors is to screen for the putative binding sites in promoter sequences of possible target genes. Thus, Breeze *et al.* (2011) suggested the NAC transcription factors to play a key role in senescence regulation, based on the discovery of an over-representation of putative NACBSs in the promoters of certain clusters of SAGs. An attempt was made to use a similar approach in this study in barley, for which the recently published draft genome (International Barley Genome Sequencing Consortium, 2012) provides new opportunities to perform genome-wide studies, even though the gene models are still preliminary. The analysis of NACBSs was extended to the more complex situation of palindromic occurrences of NACBSs. The difficulty in searching for NACBSs in promoter sequences on the basis of consensus sequences (see Table 3) is mainly due to the rather short CGT core sequence with only weak conservation of the surrounding sequences. In order to address this, seven models of palindromic sequences for the core sequences were established (Table 4; Supplementary Table S4 available at *JXB* online) with varying span widths and with varying weights on nucleotides in surrounding sequences based on the suggested NACBSs in Table 3. An over-representation of these model NACBSs could indeed be detected in 333 out of 1106 available promoter sequences of up-regulated genes during senescence, suggesting that they play a role in the regulation.

In order to substantiate the role of the putative NACBS for regulation by NAC transcription factors, the general co-expression of SAGs having an NACBS in their promoter sequence with the NAC genes was investigated. The analysis clearly showed that 71 up-regulated SAGs that had an NACBS in the promoter, compared with those that did not, exhibited strong general co-expression with senescence-associated NAC genes. Hence, not only during senescence, but in general, the occurrence of the NACBSs seems to favour co-expression of SAGs with some of the NAC transcription factor genes, which implies the importance of the NACBSs for the regulation of target genes by the NAC transcription factors. The 71 up-regulated SAGs (Supplementary Table S4 available at *JXB* online) constitute a list of possible target genes, for which further results from testing of direct interaction between promoter elements and NAC transcription factors are pending.

In conclusion, considering the large degree of conservation observed between the well-characterized *Arabidopsis* senescence processes and the results from barley presented here, the visual senescence pathway map and expression data are likely to be representative for other cereals as well. Furthermore, additional evidence of the importance of not only single NAC transcription factors but multiple members of the family for a concerted regulation of the complex senescence processes was provided. A list of up to15 barley NAC genes that are particularly interesting for this regulation process is presented. Finally, a list of 71 novel putative target genes of the NAC transcription factors is presented.

## Supplementary data

Supplementary data are available at *JXB* online.

Figure S1. Distribution of 3867 differentially expressed probes from the Agilent microarray experiment across the 35 top-bins of the MapMan annotations.

Figure S2. Correlation of gene expression levels in the Agilent microarray experiment and in qRT–PCR experiments.

Figure S3. Distributions in co-expression lists for 32 NAC genes.

Table S1. Result table after Limma analysis of microarray data.

Table S2. Annotations of genes in the MapMan top-bins of Supplementary Fig. S1.

Table S3. Annotations of genes in MapMan pathway of Supplementary Fig. 1.

Table S4. Letter probability matrices used for FIMO analysis.

Table S5. List of 71 putative target genes of NAC transcription factors.

Table S6. List of primers used in qRT–PCR experiments

Supplementary Data

## Abbreviations:

CDS: coding sequence
Ct: threshold cycle
NAC: *N*AM, *A*TAF1, *C*UC
NACBS: NAC binding sequence
qRT–PCR: quantitative reverse transcription–PCR
SAG: senescence-associated gene.
