# Supplementary material for: Members of the barley NAC transcription factor gene family show differential co-regulation with senescence-associated genes during senescence of flag leaves
Source: J Exp Bot. 2014 Feb 24;65(14):4009–22. doi: 10.1093/jxb/eru046 (PMC4106437; doi:10.1093/jxb/eru046)
Supplement: Supplementary Data [file supp_65_14_4009__index.html]

Members of the barley NAC transcription factor gene family show differential co-regulation with senescence-associated genes during senescence of flag leaves — Members of the barley NAC transcription factor gene family show differential co-regulation with senescence-associated genes during senescence of flag leaves — Supplementary Data 

# Members of the barley NAC transcription factor gene family show differential co-regulation with senescence-associated genes during senescence of flag leaves

## Supplementary Data

Data files

**Files in this Data Supplement:**

- Supplementary Data - Supplementary Data
- Supplementary Data - Supplementary Data
- Supplementary Data - Supplementary Data
