# Supplementary material for: Members of the barley NAC transcription factor gene family show differential co-regulation with senescence-associated genes during senescence of flag leaves
Source: J Exp Bot. 2014 Feb 24;65(14):4009–22. doi: 10.1093/jxb/eru046 (PMC4106437; doi:10.1093/jxb/eru046)

Title: Members of the barley NAC transcription factor gene family show differential co-regulation with senescence-associated genes during senescence of flag leaves.

Authors: Michael W. Christiansen and Per L. Gregersen

Figure S1. Distribution of signals from 3867 probes from the Agilent microarray experiment across the 35 top-bins of the MapMan annotations. The probes were selected as representing differentially expressed genes in senescing barley flag leaves. Blue indicates down-regulation and red up-regulation.

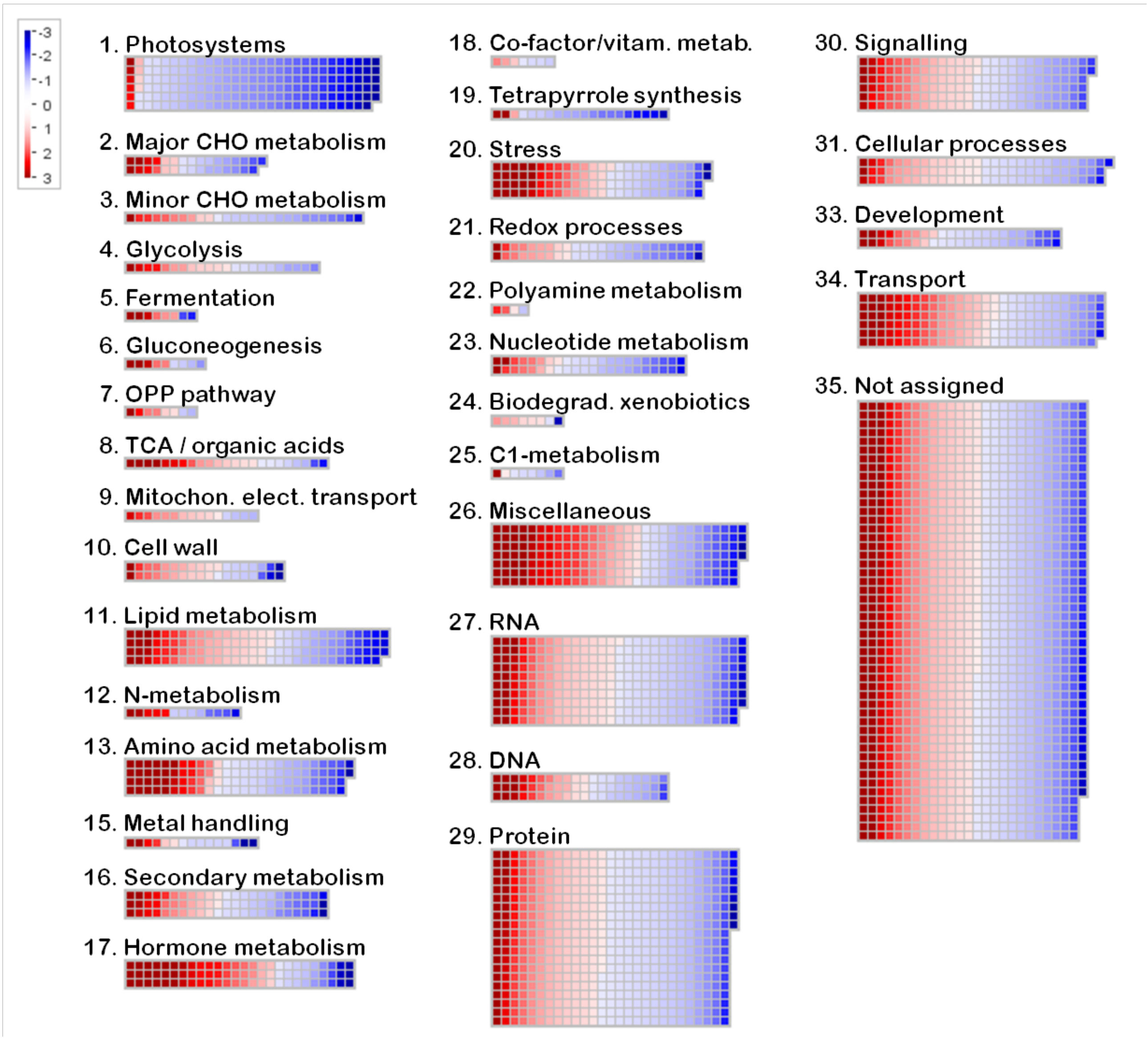

Figure S2. Correlation of gene expression levels in the Agilent microarray experiment and in qRT-PCR experiments. A) NAC genes – qRT-PCR data are from Christensen *et al.* (2012). Closed symbols indicate significantly differentially expressed genes. B) Selected senescence-associated genes (SAGs), both experiments from this work.

A) - NAC genes

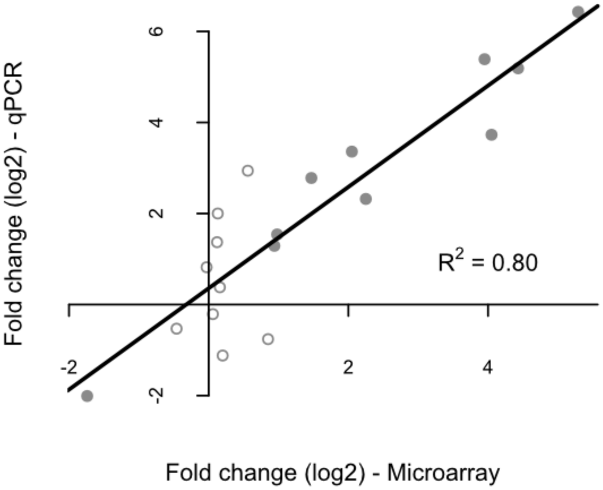

B) - SAGs

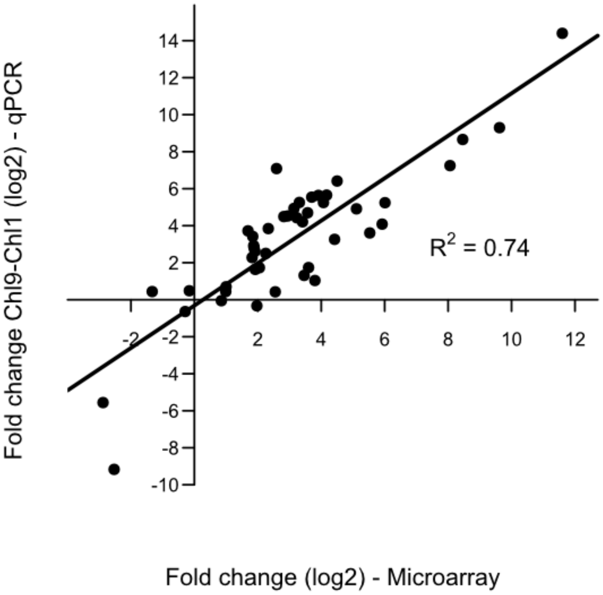

Figure S3. The distribution in co-expression lists for 32 NAC genes of the 71 senescence-upregulated genes having NACBS-8 in their promoter (gray) and 1181 up-regulated genes without any NACBS (white). The lower the rank, the higher the co-expression with the indicated NAC genes (Mochida *et al.* 2011).

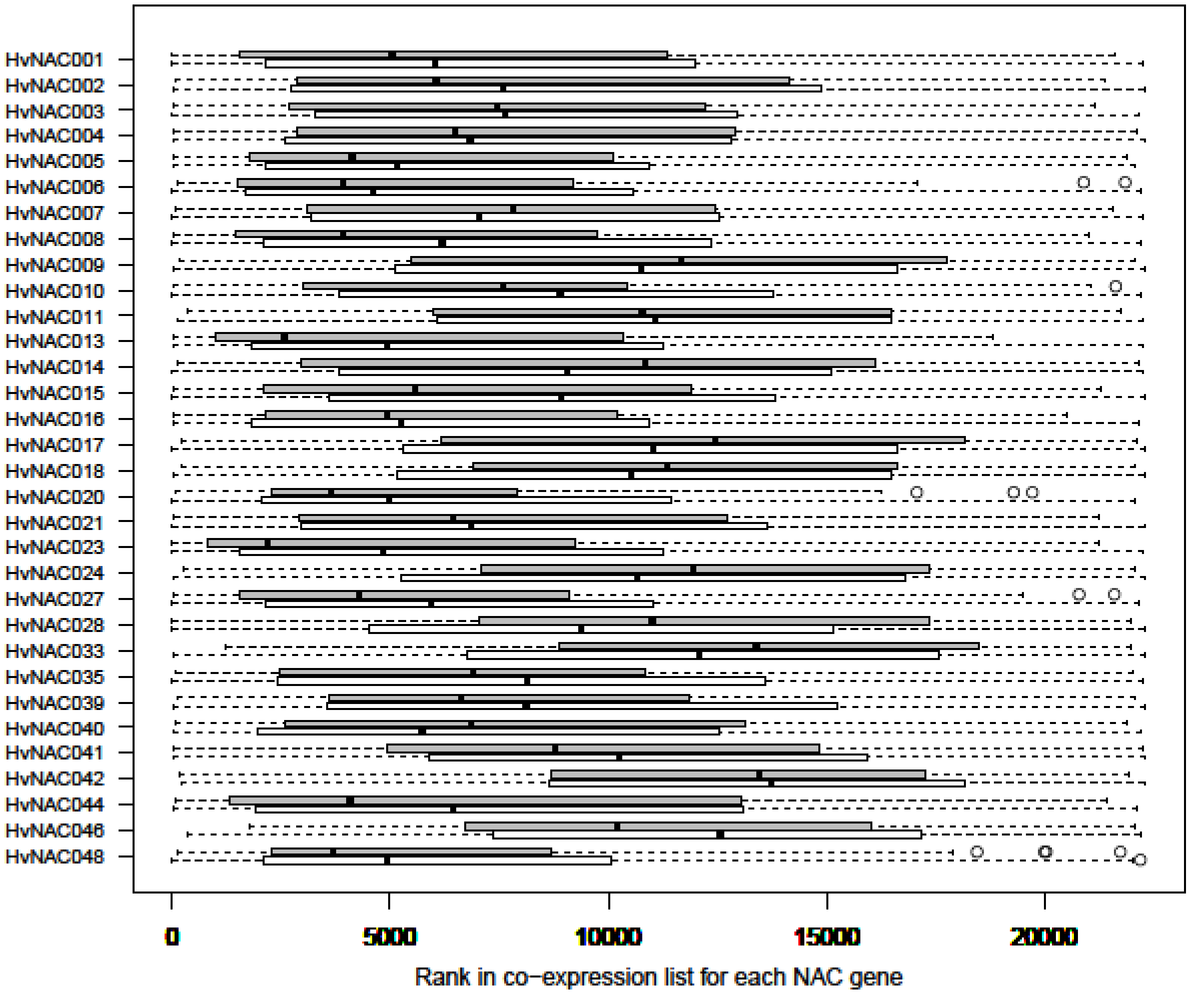

Supplement: Supplementary Data [file supp_eru046_jexbot116301_file001.pdf]
