# Supplementary figures and images for: Members of the barley NAC transcription factor gene family show differential co-regulation with senescence-associated genes during senescence of flag leaves
Source: J Exp Bot. 2014 Feb 24;65(14):4009–22. doi: 10.1093/jxb/eru046 (PMC4106437; doi:10.1093/jxb/eru046)

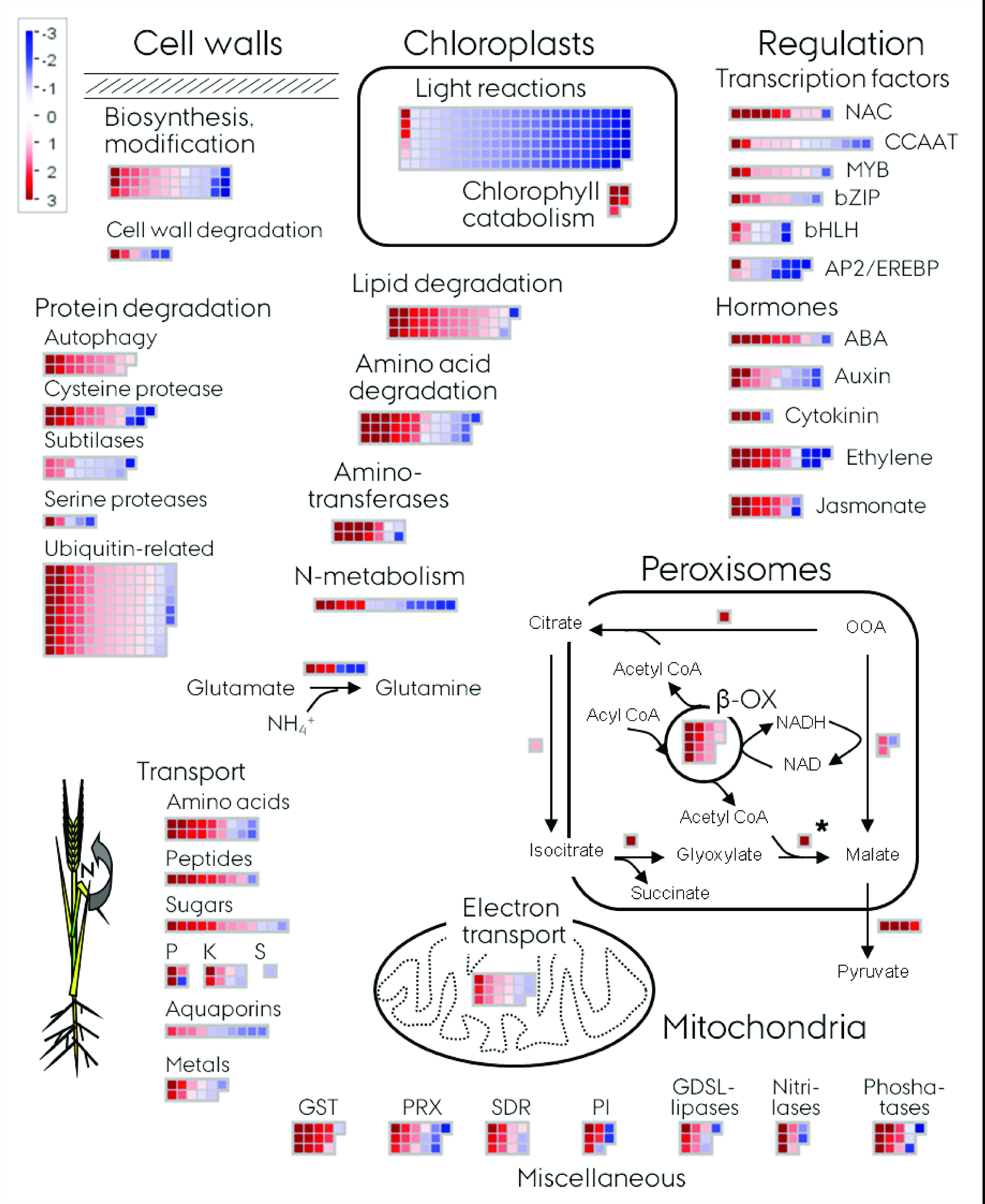

Supplement: Supplementary Data [file supp_eru046_jexbot116301_file003.tif]
